# Dataset on cellular and geo-spatial information of a 10 km distance along Akure-Ilesha road

**DOI:** 10.1016/j.dib.2018.12.035

**Published:** 2018-12-17

**Authors:** Reuben.S. Diarah, Samson A. Oyetunji, Christian O. Osueke, A.O. Onokwai

**Affiliations:** a Landmark University Omu-Aran, Kwara State, Nigeria; b The Federal University of Technology Akure, Ondo State, Nigeria

## Abstract

This dataset contains cellular and geo-spatial information of a 10 km distance along Akure-Ilsha road in Ondo state, Nigeria. The data was acquired using a designed data acquisition system which was kept inside golf3 vehicle interfaced with Acer laptop, the data was acquired as the vehicle moves from the reference point (*7.39919, 5.05944 )* to its destination point (*7.32818, 5.10836)*, it harvests GSM signal Strengths from a base station in intervals with its time, latitude and longitude simultaneously as the vehicle moves along the travelled rout; the data acquired shows the variation of signal strength against distance along the road from one base station to another in the travelled path. The raw data of this work is hosted in the Mendeley repository DOI:10.17632/tmksc8mkt8.1

**Specifications table**

**Table:** 

Subject area	*Electrical and Electronic Engineering.*
More specific subject area	*Communication.*
Type of data	*Table, image, graph, figure.*
How data was acquired	*The data was collected using the designed data acquisition system, the data acquisition device is shown in*image 2*,it is made up of GSM and GPS module that enable the device to receive the GSM Signal strength and Latitude and longitude along the travelled path, data from the designed acquisition system* was correlated with data obtained from a standardize equipment to establish the integrity of the data which gave a correlation of 0.98. The designed acquisition system was kept inside golf3 vehicle interfaced with Acer laptop, the data was acquired as the vehicle moves from the reference point to its destination point.
Data format	*Raw data.*
Experimental factors	*Sim800 module was used to access cellular network and Neo-6 GPS Module was used to get geo-spatial coordinates (latitude and longitude.)*
Experimental features	*The designed data acquisition system have the following features, GSM Module,Neo-6 GPS Module, memory card Atmel 328, Ardinuo uno and a control that controls all these together.*
Data source location	*Akure-Ilesha road (7.39919, 5.05944 and 7.32818, 5.10836) Nigeria.*
Data accessibility	*The data is attached with this submission and can also be downloaded via*https://data.mendeley.com/datasets/tmksc8mkt8
Related research article	Pierpaolo .Salvo et al. Heterogeneous cellular and DSRC networking for Floating Car Data collection in urban areas, Vehicular Communications, Volume 8 (2017) doi.org/10.1016/j.vehcom.2016.11.004

**Value of the data**

• This dataset will enable research to go on saving researchers time of coming to this location for real time data capturing of signal strengths along this path.
• The dataset will be very useful in analysis of signal strength and handoff along this route.
• This will reduce cost of carrying out similar kind drive test along this road.
• The signal strengths collected along this was correlated with a standardized equipment/App to verify its integrity.

## 1 Data

The set of data that was collected using this designed data acquisition system includes the signal strengths, the geo-spatial information (Latitude and Longitude), date, time and the type of network This dataset was collected using a designed data acquisition system that was kept inside a golf3 vehicle as the vehicle moves from its reference point (latitude 7.39919, longitude 5.05944) to its destination and latitude (7.32818, longitude 5.10836) along Akure-Ilesha road, Ondo, State, Nigeria.

**Image 1:**
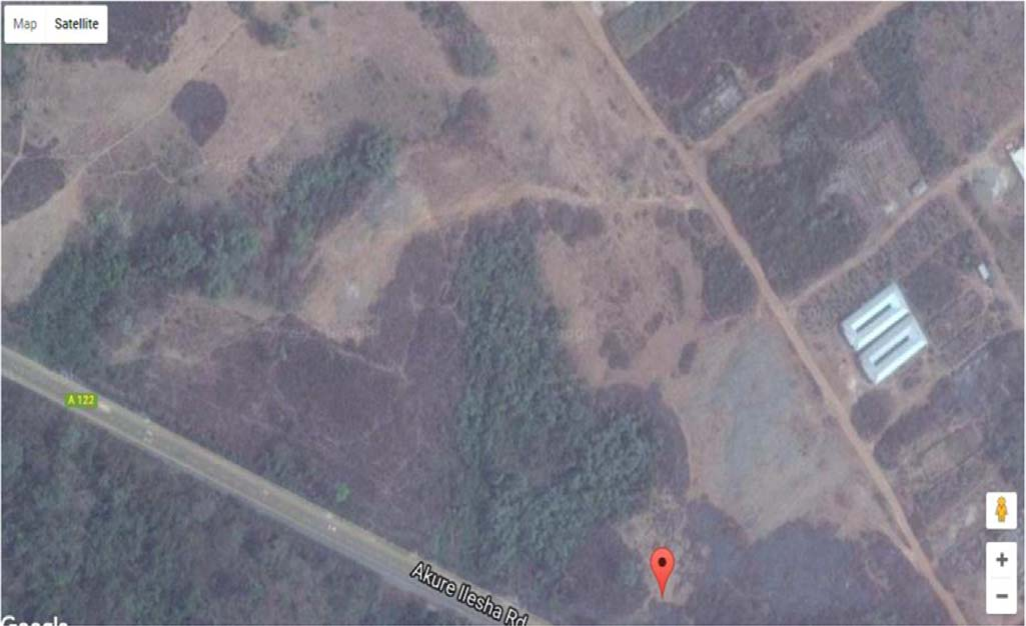
: The study area Akure-Ilesha road.

**Image 2:**
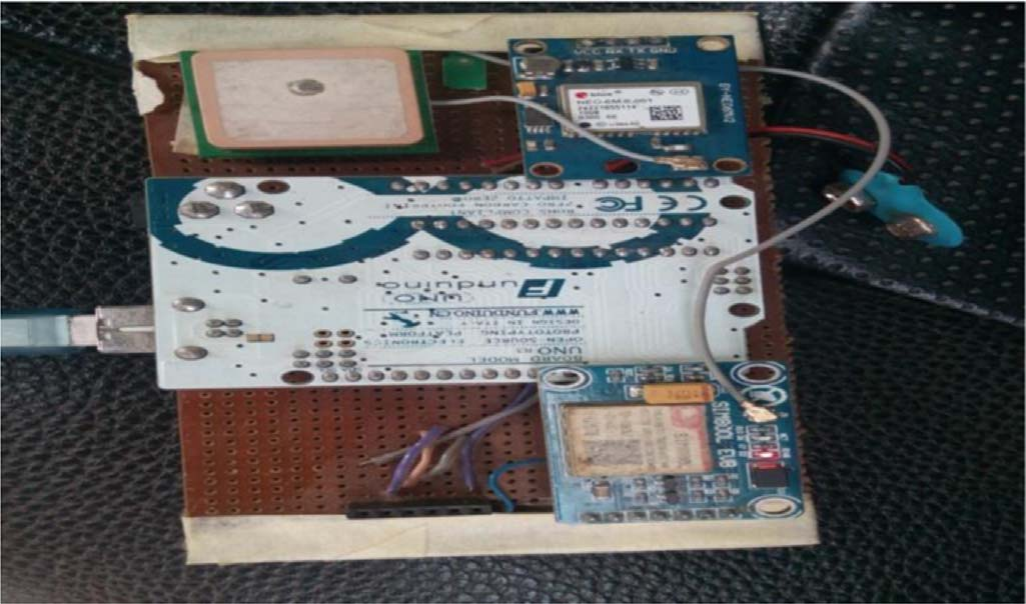
: Pictorial view of the Designed Data acquisition system.

Image 1, Image 2 shows the study area where this data was acquired and pictorial view of the data acquisition system. Table 1.1 shows the data collected along the road during drive test as the vehicle moves from its reference position, the signal strength along the travelled path was recorded in interval at different times for a distance of 10 km, from the data that was harvested from the travelled path, the graph of signal strength against distance for each 2 km drive test was plotted for the 10 km trip along Akure-Ilesha road.

**Table 1.0:** Data received from designed data acquisition.

Report	sys_date	sys_time	net_op_name	net_op_code	lac_tac	cid	rssi	Latitude	Longitude
0	2018/7/9	9:15:56	MTN NG	62130	20586	25767	−15	7.39919	5.05944
1	2018/7/9	9:15:58	MTN NG	62130	20586	25767	−15	7.39919	5.05944
2	2018/7/9	9:15:59	MTN NG	62130	20586	25767	−41	7.39919	5.05944
3	2018/7/9	9:16:00	MTN NG	62130	20586	25767	−41	7.39919	5.05944
4	2018/7/9	9:16:01	MTN NG	62130	20586	25767	−41	7.39919	5.05944
5	2018/7/9	9:16:02	MTN NG	62130	20586	25765	−29	7.39919	5.05944
6	2018/7/9	9:16:03	MTN NG	62130	20586	25765	−29	7.39918	5.05943
7	2018/7/9	9:16:04	MTN NG	62130	20586	25765	−29	7.39918	5.05943
8	2018/7/9	9:16:05	MTN NG	62130	20586	25765	−29	7.39918	5.05943
9	2018/7/9	9:16:06	MTN NG	62130	20586	25765	−29	7.39917	5.05943
10	2018/7/9	9:16:07	MTN NG	62130	20586	25765	−29	7.39915	5.05943
11	2018/7/9	9:16:08	MTN NG	62130	20586	25765	−29	7.39915	5.05943

**Table 1.1:** Data collected along the road during drive test.

net_op_name	net_op_code	mcc	mnc	lac_tac	cid	rssi	Lat	long	distance	mrssi
MTN NG	62130	621	30	20586	25767	−15	7.39919	5.05944	0	
MTN NG	62130	621	30	20586	25767	−15	7.39919	5.05944	0	
MTN NG	62130	621	30	20586	25767	−41	7.39919	5.05944	0	
MTN NG	62130	621	30	20586	25767	−41	7.39919	5.05944	0	
MTN NG	62130	621	30	20586	25767	−41	7.39919	5.05944	0	
MTN NG	62130	621	30	20586	25765	−29	7.39919	5.05944	0.001566	
MTN NG	62130	621	30	20586	25765	−29	7.39918	5.05943	9.49E−05	−30.14
MTN NG	62130	621	30	20586	25765	−29	7.39918	5.05943	9.49E−05	−32.14
MTN NG	62130	621	30	20586	25765	−29	7.39918	5.05943	0.001111	−34.14
MTN NG	62130	621	30	20586	25765	−29	7.39917	5.05943	0.002222	−32.43
MTN NG	62130	621	30	20586	25765	−29	7.39915	5.05943	0	−30.71
MTN NG	62130	621	30	20586	25765	−29	7.39915	5.05943	0	−29
MTN NG	62130	621	30	20586	25765	−29	7.39915	5.05943	0.001566	−29
MTN NG	62130	621	30	20586	25765	−29	7.39916	5.05944	0.001111	−29
MTN NG	62130	621	30	20586	25765	−29	7.39915	5.05944	0.001569	−29
MTN NG	62130	621	30	20586	25765	−29	7.39914	5.05943	0	−29

Table 1.0 shows part of the data received from the designed data acquisition system over a distance of 10 km for signal strength and distance, the full details of the data can be accessed through [1].

## 2 Experimental design, materials, and methods

The data acquisition system was designed to harvest the signal strengths and the latitude and Longitude of the travelled path so that these data can be used to estimate the journey of the vehicle, the following device were used in the design of the data acquisition system:

• Neo-6 GPS Module.
• Atmel 328P-pu microcontroller.
• Sim800 GSM Module.
• Memory card Module.
• Ardinuo uno ; and
• A control code

**Fig. 1:**
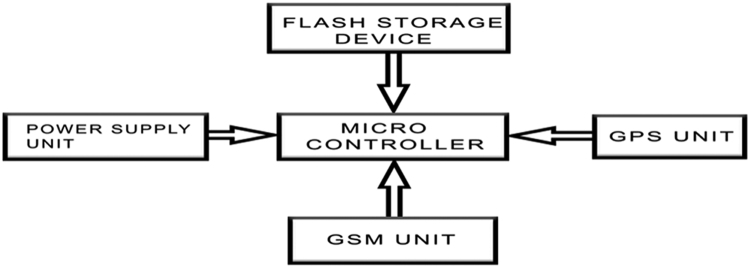
Block diagram of designed data acquisition system.

The Neo-6 GPS Module is used in acquiring the geo-spatial(Latitude and longitude) of every point and the Sim800 GSM module is used to harvest the signal strengths along the travelled path as the vehicle moves from one location to the other, the Atmel 328 microcontroller with a control code controls the whole process of the system (Fig. 1).

**Fig. 1.1:**
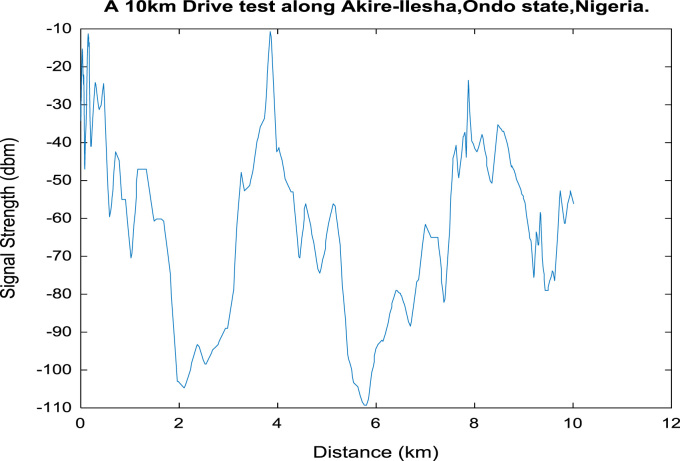
Plot of signal strength against distance in 2 km drive test.

**Fig. 1.2:**
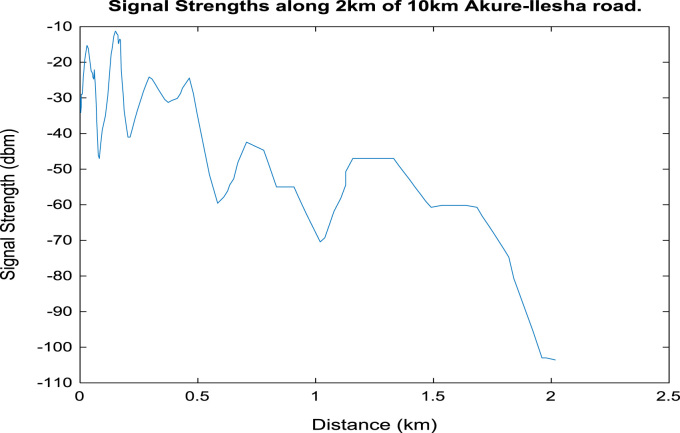
Plot of signal strength against distance in 2 km drive test.

**Fig. 1.3:**
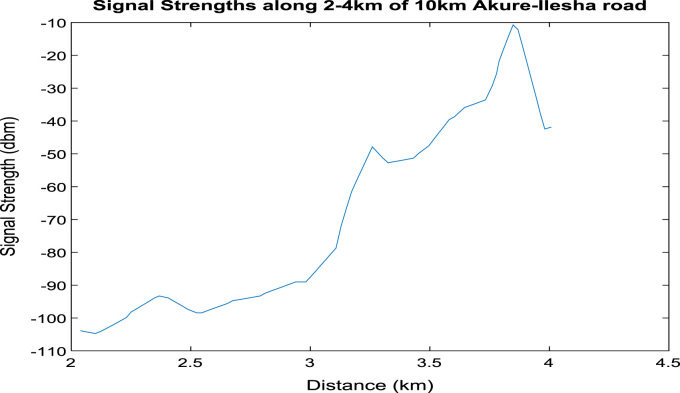
Plot of signal strength against distance in 2 km drive test.

**Fig. 1.4:**
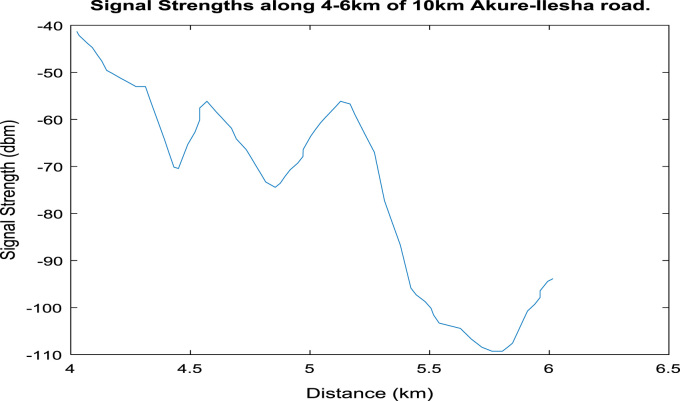
Plot of signal strength against distance in 2 km drive test.

**Fig. 1.5:**
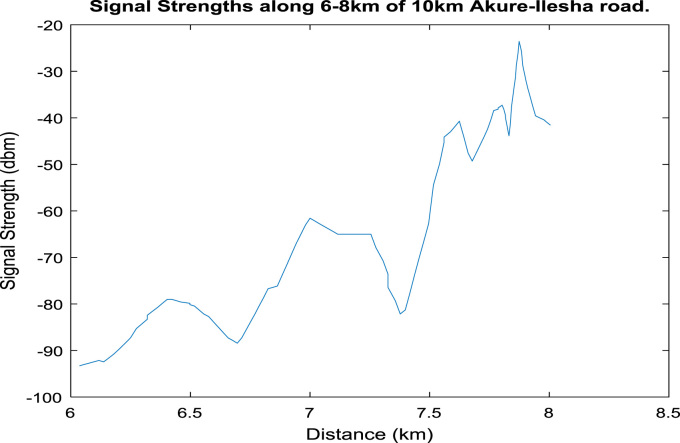
Plot of signal strength against distance in 2 km drive test.

**Fig. 1.6:**
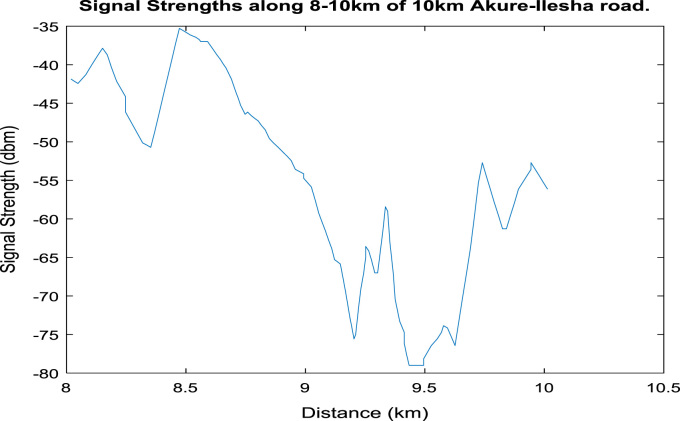
Plot of signal strength against distance in 2 km drive test.

Fig. 1.1, Fig. 1.2, Fig. 1.3, Fig. 1.4, Fig. 1.5, Fig. 1.6 shows the plot of signal strength against distance for the each 2 km distance in the 10 km trip along Akure-Ilesha
