# Supplementary figures and images for: Dataset on cellular and geo-spatial information of a 10 km distance along Akure-Ilesha road
Source: Data Brief. 2018 Dec 17;22:530–6. doi: 10.1016/j.dib.2018.12.035 (PMC6322077; doi:10.1016/j.dib.2018.12.035)

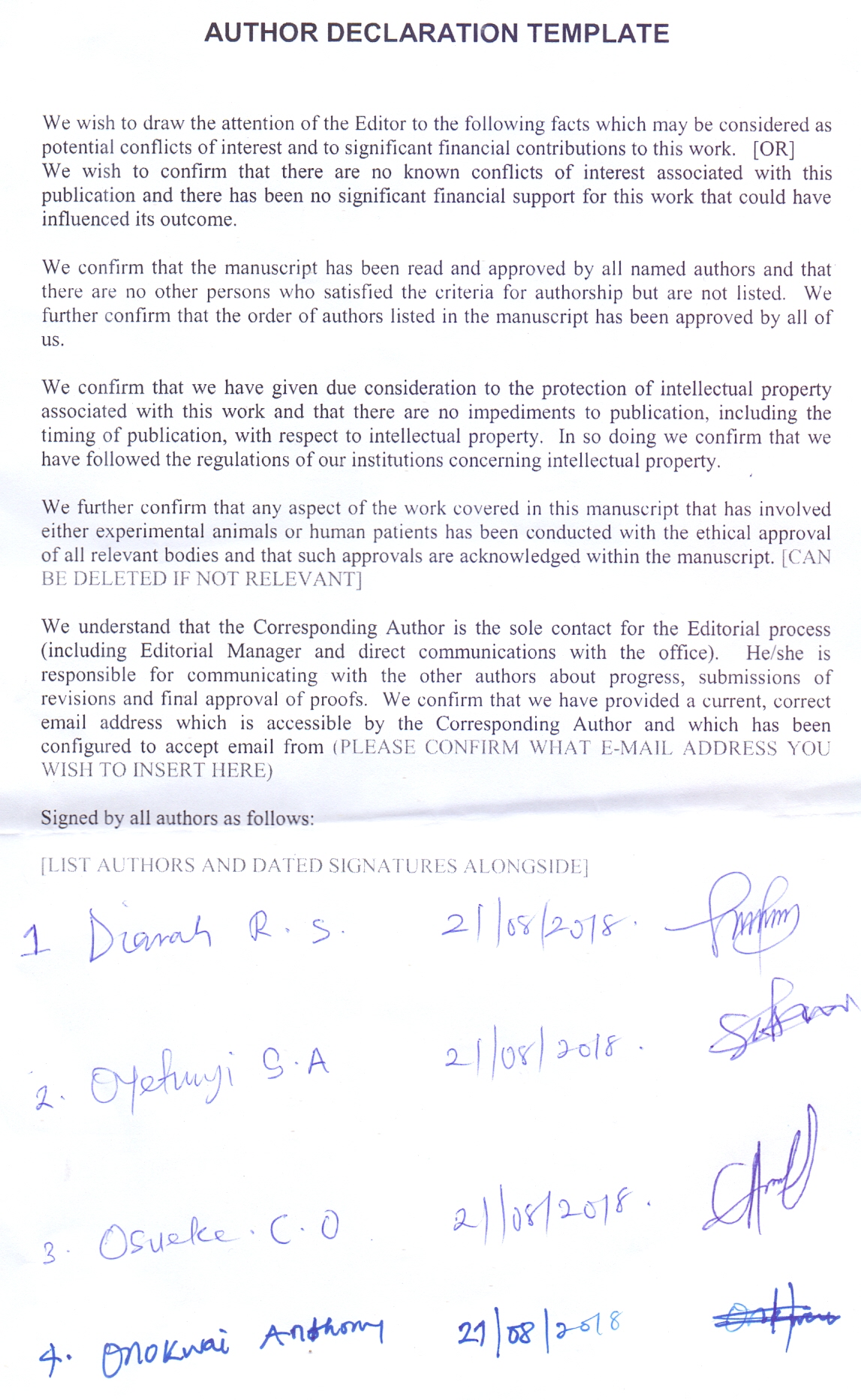

Supplement: Supplementary file 1 — Supplementary material [file mmc1.docx]
